# Predictive but not emotional value of Pavlovian stimuli leads to pavlovian-to-instrumental transfer

**DOI:** 10.1016/j.bbr.2016.12.022

**Published:** 2017-03-15

**Authors:** Stephens Jeffs, Theodora Duka

**Affiliations:** School of Psychology, University of Sussex, Brighton, BN1 9QH, UK

**Keywords:** Emotion, Expectancy, Pavlovian-to-instrumental transfer, Evaluative-conditioning

## Abstract

Reward-predictive stimuli augment instrumental reward-seeking in humans, an effect denoted Pavlovian-to-instrumental transfer (PIT), but the role of differential reward value of these stimuli and of emotional conditioned responses in PIT remains unknown.

Fifty one participants experienced a Pavlovian phase that associated two stimuli with either 10p (CS10) or 50p (CS50). Next, participants underwent instrumental training for two responses reinforced with either 10p or 50p. Finally, the transfer phase continued as had instrumental training, now in the presence of the Pavlovian stimuli.

Participants were dichotomised as aware/unaware according to their expectancy awareness of the CS → outcome associations. Only aware participants demonstrated PIT (increased choice and number of responses on the 10p and 50p response key in the presence of CS10 and CS50 respectively), yet both aware and unaware groups rated the 50p stimulus as more pleasant than the 10p stimulus.

These findings suggest that expectancy of reward is necessary for PIT; however, emotional conditioned responses appear not sufficient to influence PIT. Future research should attempt to manipulate emotional conditioned responses in a PIT context, to test the sufficiency of reward expectancy in PIT.

## Highlights

• Pavlovian stimuli (CSs) of different reward value induce outcome specific transfer.
• Knowledge of the predicted outcome appears necessary for the transfer to occur.
• Emotional response to CSs occurs without knowledge of predicted outcome.
• Emotional response to CSs is not sufficient to elicit transfer.

## 1 Introduction

Multiple theories of reward-seeking converge on a causal role of stimuli paired with positive reward-induced states in activating behavioural processes [1]. Such stimuli prime reward states in a similar manner to primary rewards themselves [2], elicit approach behaviour to reward-associated targets [3], and activate processes attributing incentive salience to reward procurement [4]. While these theories differ subtly in their arguments concerning the role of such stimuli in reward-seeking, the theories each predict that stimuli increasingly activate systems representing the motivational value of the outcome.

In order to ensure that any effect of a stimulus (S) on a response (R) is due to an outcome (O) representation, it is necessary to separate the training of S → O from R → O. Under such conditions, any effect of a stimulus on a response must occur indirectly via an outcome representation, as the direct S → R association has never been explicitly reinforced [5]. Any influence of a stimulus on a response under such separated training conditions is termed Pavlovian-to-instrumental transfer [PIT, 6].

PIT can be divided into two forms − *specific* and *general*. Specific-PIT is evidenced by a response bias towards the specific outcome predicted by the stimulus [7], [8], [9]. General-PIT, in contrast, is evidenced by a general augmentation of responding in the presence of an excitatory stimulus regardless of whether stimulus and response share an outcome [10], [11]. While specific-PIT is argued to rely on a cognitive representation of outcome identity [5], general-PIT may be more sensitive to a hedonic representation of outcome value [12], [13].

Human PIT research supports the notion that transfer, in either form, only occurs in participants who expect a specific outcome after seeing its associated stimulus [8], [14], [15], [16], [17]. But this extant literature has also shown that participants who display a conditioned expectancy response also display a conditioned emotional response. Thus expectancy and emotion have been confounded, making it difficult to separate their relative importance in PIT.

However, a meta-analysis by Hofmann and colleagues [18] reported that emotional conditioned responses can be elicited by stimuli in the absence of an expectancy response. While Hofmann and colleagues’ analysis demonstrated that these emotional conditioned responses were stronger in participants possessing such expectancy awareness, effect sizes in “unaware” participants were “still reliably greater than zero” [18,p. 406]. These conditioned emotional responses have been shown to track the hedonic value of their associated outcomes [19], [20], [21], further suggestive of the causal predictive role of emotional conditioned responses in reward-seeking. But the behavioural consequences of conditioned emotional responses in the absence of conditioned expectancy responses have not been tested under PIT conditions.

In light of these gaps in understanding, the present experiment was devised to test how differential reward value can lead to outcome specific transfer as determined by the choice of the relevant response (the one that will produce the predicted outcome), but also whether the different reward value will induce differential degree of motivation as measured by the response rate (the number of presses) on the outcome relevant key. Another important aim of the study was to examine whether emotional conditioned responses (ECR; ratings of pleasantness induced by the stimulus associated with high reward value) also influence reward-seeking behaviour. To the authors' knowledge there is no study in the literature that has directly tested the possible differential effects of ECR to Pavlovian stimuli on PIT. The hypothesis we put forward is that the effects of different reward value on PIT will depend on the explicit knowledge of stimulus outcome contingencies, whereas the emotional responses generated by stimuli of different value will be independent of the contingency knowledge; the open question remains whether the isolated hedonic value representation elicited by a stimulus is sufficient for specific-transfer to occur. Thus the present experiment exploited the phenomenon whereby a stimulus paired with an emotionally salient outcome activates ECR without explicit activation of expectancy conditioned responses [18]. Money was used as the outcome to ensure that behaviour was unbiased by acute substance ingestion [22], [23], [24].

## 2 Method

### 2.1 Participants

54 University of Sussex students (23 males), mean age 21.7 years (range 18–37), were recruited via an online participant database and compensated for their time financially or with course credit. Participants gave written consent before beginning the study, with ethical approval being granted by the University of Sussex Life Sciences ethics committee. Inclusion criteria were that English was their first language, and that they were in a state of good health, whereas exclusion criteria were that they were currently taking prescription medication (excluding the contraceptive pill), reported having been diagnosed with a mental illness, smoked more than five cigarettes per week, or reported a gambling problem.

### 2.2 Materials

#### 2.2.1 Behavioural tasks

**Fig. 1:**
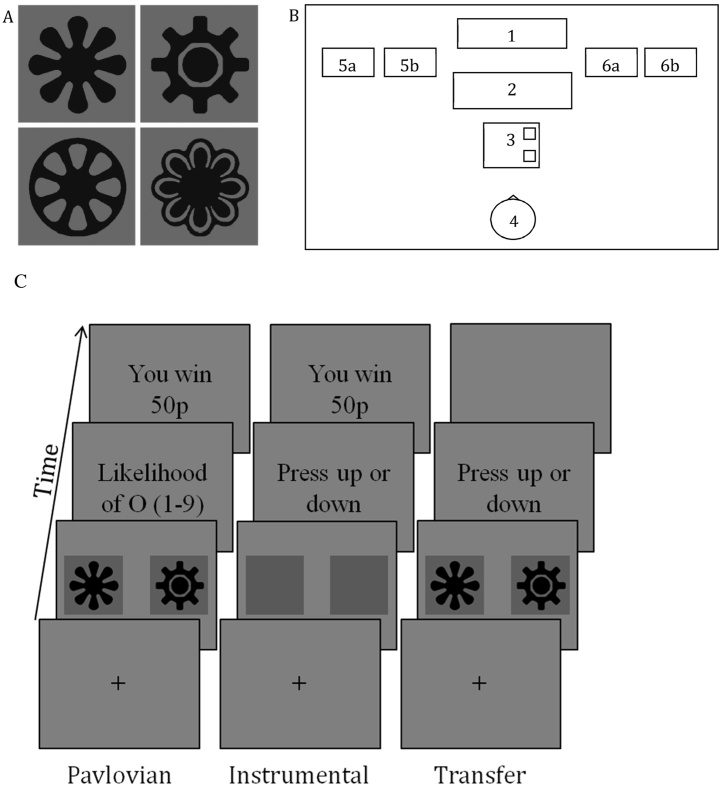
Stimuli used in Pavlovian and transfer phases of the experiment [A], layout of apparatus within cubicle, aerial view [B] and Diagram of PIT procedure [C]; Pavlovian. Trials began with a fixation cross for duration 1s. This was then replaced by a stimulus pair (CS; 3 s). The outcome (O) expectancy question then appeared with anchors “1 = 10p, 5 = don't know, 9 = 50p”. Upon response a grey screen appeared (1s; not shown), followed by an instruction to “Press the spacebar to find out how much you have won” (not shown). Immediately after pressing the spacebar participants were informed of the outcome (10p/50p; 2s), which was contingent solely upon the CS displayed (and so not dependent on a correct response to the expectancy question). Instrumental. Trials began with a fixation cross (1s). Then a pair of identical dark grey squares (distinguishable from the lighter background) appeared (2s). This was followed by a screen prompting participants to select a response, after which came a reinforcement screen (10p/50p; 2s). Transfer. Transfer was similar to instrumental training. However, grey squares appeared in only ⅓ of trials, with the remaining ⅔ split equally between CS10 and CS50 trials; also the reinforcement screen was replaced by a blank screen (1s). 1 = LCD screen; 2 = keyboard; 3 = response box; 4 = participant; 5a/b = 10p/50p coin box; 6a/b = participant’s 10p/50p winnings box. Note that keyboard and response box were both placed in the location of 3–keyboard during Pavlovian training, response box during instrumental training and transfer.

The PIT task was run on a PC using E-Prime v1.2 software [25]. The display background during the task was always grey. Stimuli (see Fig. 1A) were presented at a size of 10.2 cm^2^ at a resolution of 1280 × 1024 pixels. During Pavlovian training responses were recorded using a standard QWERTY keyboard. During instrumental training and transfer the keyboard was replaced by a five-button response box. Buttons were aligned along the sagittal axis; only the first and fifth buttons were active, and were coloured blue with a black arrow pointing towards or away from the participant, respectively, to indicate their activation. Throughout the experiment to the left and right of the response equipment were two metal boxes with their lids open. Inside the left boxes were either 64 ten pence coins or 64 fifty pence coins. The right boxes were initially empty, but were labelled with “Your 10p box” or “Your 50p box” (see Fig. 1B for a schematic of the apparatus layout).

### 2.3 Design and procedure

#### 2.3.1 Pavlovian conditioning

The PIT task comprised three phases (see Fig. 1C for a diagram of participants’ experience of the PIT task, including event durations). First was a Pavlovian conditioning phase that associated one stimulus out of the four (see Fig. 1A) with 10p (stimulus A), and another stimulus with 50p (stimulus B), both with 100% contingency with the monetary outcome. The two remaining stimuli (stimulus X and Y) were presented horizontally adjacent to stimulus A and B resulting to a 50% contingency with each outcome, and so were non-predictive of either outcome. The inclusion of these non-predictive stimuli was informed by a similar method used by Hogarth et al. [8], who demonstrated that the added complexity afforded by the stimuli X and Y delayed the learning of the outcome contingencies and allowed a better differentiation between aware and unaware participants. Each trial presented either A10 or B50, combined with either X10/50 or Y10/50, providing the trials with the CS10 and CS50 stimuli (we keep this nomenclature throughout the paper when we are referring to our data). The roles of the four stimuli, and relative position (left or right), was counterbalanced, order of presentation was random.

Participants read the following instruction screen before beginning the task:

The following task is made up of trials where you can win 10 pence and 50 pence. Each trial will begin with a fixation cross (+) in the centre of the screen, which you should look at. Then two pictures will appear. Immediately afterwards you will be asked to rate how likely you think you are to win 10p or 50p. You will then be prompted to press the spacebar to find out how much you have won. The amount you win is dependent upon which pictures were shown on the screen. Press the spacebar to begin.

There were 128 trials in total, 64 10p trials, 64 50p trials, divided into 8 blocks of 16. Separating CSs and outcome screens was an expectancy question: “How likely are you to win 10p or 50p? 1 = 10p, 5 = don't know, 9 = 50p” (although it was also explained to participants verbally that they could use all of the number keys 1–9). After the expectancy question was a grey screen and spacebar press before the outcome, e.g. “You win 10p”.

After each block participants were shown a screen that detailed their total winnings for that block (always £4.80), and were instructed to move the amount from the left-hand boxes into the right-hand boxes. After one block the experimenter re-iterated instructions verbally, to ensure task comprehension, before leaving the participant to complete the remaining trials alone.

Expectancy ratings were used to classify participants in those with the ability to discriminate between the two stimuli associated with different values (50p versus 10p; named “aware”) and in those who were unable to discriminate between the two stimuli associated with different values (50p versus 10p; named “unaware”). For this purpose the 16 expectancy ratings from the final block of Pavlovian training were used. Participants were classified as ‘aware’ if they had significantly lower expectancy rating for the CS predicting 10p than for the CS predicting 50p (given that ‘1 = 10p, 9 = 50p’ during the task).

#### 2.3.2 Emotional ratings emotional conditioned response; ECR

Immediately after Pavlovian conditioning, participants were asked to rate their emotional evaluations of each stimulus. The stimuli (A, B, X, and Y, parts of the compound CS10 and CS50) were presented individually in random sequence with a single rating question − “How pleasant do you find this picture?" or ‘How anxious does this picture make you feel?" (counterbalanced) − and response scale below it. Participants were instructed to “Press a number key between 1 and 9 to indicate the strength of your feeling, 1 = not at all, 9 = extremely’. Each stimulus remained on screen until a response was made, at which point a blank screen appeared for a random duration of 2-2.5s, before the next stimulus and question was presented.

#### 2.3.3 Instrumental training

Having given their emotional evaluations participants took a five minute break before seeing written instructions detailing the instrumental task:

In this session, by pressing the up or the down button on the response box, you will be able to win either 10 pence or 50 pence. Pressing one button wins 10p, pressing the other wins 50p. Sometimes you will win the money, sometimes you will win nothing. Trials will start with a fixation cross (+), which you should look at. The cross will then be replaced by two squares. Following this you will be asked to press one of the buttons. You will only win if you press repeatedly while the prompt appears on the screen, and only press one button within each trial. Press either button to begin.

When participants press successfully the screen appeared with the written information “you win 10p (or 50p). If participants did not press or pressed unsuccessfully (i.e. not repeatedly or on both keys, only a blank screen was presented. There were 100 trials in total, divided into 5 blocks of 20. Each block ended with a screen displaying participants’ winnings for that block (the amount was response-contingent), and asked them to move the specified amount into their winnings box. The experimenter re-iterated the instructions verbally after 20 trials to ensure task comprehension, before leaving the participant to complete the task alone. Presses on the 10p button (R10) were reinforced with a 50% contingency, while presses on the 50p button (R50) carried a 10% contingency. This ensured that the utility of each button was identical and so discouraged a bias towards one response or the other. Association between button and monetary outcome was counterbalanced.

To encourage repeated pressing within each trial, reinforcement was contingent upon a customised variable interval 2.75 s schedule (VI2.75). Participants were required to press at least twice within a 1 s window of random onset (minimum 1.5s) after the presentation of the button-press prompt. This alteration to the traditional VI setup, where a single response after a time delay is sufficient to receive reward, ensured that participants pressed multiple times, rather than simply pressing once towards the end of each trial. Responses during this phase represent the baseline responding (before the CSs were presented; transfer phase).

#### 2.3.4 Transfer

The transfer phase was integrated with the instrumental training phase to appear as a continuation of the same task. However, to balance the need to preclude new learning while maintaining behavioural responses, participants saw the following instruction screen at the start of the transfer phase:

Now you will continue to earn money as before, but you will only be told how much at the end of the session. Sometimes the pictures you saw earlier will be presented. Press either button to continue.

Transfer proceeded similarly to instrumental training. However, the reinforcement screen was replaced by a blank screen. Furthermore, ⅓ of trials were replaced with the Pavlovian stimulus pair CS10, another ⅓ with the stimulus pair CS50, with the final ⅓ retaining the grey squares to measure blank trials (presented along the CSs) responding. There were 96 trials in total, split into 2 blocks of 48. Thus each trial was presented 16 times per block. Transfer blocks were separated by a screen announcing “Halfway. Press either button to resume the task” the duration of which was participant controlled. The entire PIT procedure took approximately 60mins to complete − 30 min for Pavlovian training, 30 min for the instrumental and transfer phases combined. Immediately after the experiment, the experimenter conducted a debriefing interview to gain qualitative information about participant awareness of Pavlovian and instrumental contingencies.

Immediately following the transfer phase, the experimenter conducted a debriefing interview to gain qualitative information about participant awareness of Pavlovian and instrumental contingencies. The experimenter presented each stimulus (A, B, X, Y), in isolation, and asked the participant: “in the first part of the experiment, when you were asked to rate how likely you were to win 10p or 50p, what did you think you’d win when you saw this picture?”

### 2.4 Statistical analyses

3 participants were excluded following the experiment for failing to receive reinforcement on the R50 during instrumental training leaving a total of 51 participants (21 male) mean age 21.5 years (range 18–37),

Thirty two participants were found to be “aware” whereas nineteen participants were found to be “unaware” as classified in the present study.

Behaviour during instrumental training and transfer was operationalised as the percentage of trials where participants chose to respond, hereafter Response Choice, as well as the number of button presses per second during the button-press prompt, hereafter Response Rate. Response Choice during instrumental training was calculated as percentage of trials on which participant initiated a response; calculations were separated for R10 key (50 trials in total) and R50 key (50 trials in total) responses.

Response Choice in the transfer phase was calculated as percentage of trials on which participant initiated a response; calculations were separated for when CS10, CS50 and Blank stimulus (baseline) was presented (32 trials per stimulus) and for responses on R10 or R50 key (e.g.% response choice CS10/R10 and CS10/R50)

Response Rate was calculated by taking the total number of presses, before dividing by the total response duration per trial category (e.g. 4 s × 32 trials for CS10, CS50 and blank stimulus); again calculations were separated for responses on R10 and R50 key (e.g. response rate CS10/R10 and CS10/R50. On either variable, a specific-PIT effect would be demonstrated by increased responding on the button congruent versus incongruent with the displayed CS, i.e. R50|CS50 > R50|CS10.

In order to examine the relationship between the emotional responses to Pavlovian cues and the motivation to respond for the cue of higher value, Pearson’s bivariate correlations were performed separately for the aware and unaware group between the differences in emotional ratings to CS50 over CS10 and the differences in Rate of responses to CS50 over CS10 (on the congruent response keys) during transfer.

Data were analysed using SPSS 20.0. Bonferroni method was used to control for Type I error inflation due to multiple comparisons. Greenhouse-Geisser method was used to adjust degrees of freedom due to non-sphericity in repeated-measures analyses. Effect sizes for ANOVAs, partial eta squared, were calculated by SPSS.

## 3 Results

### 3.1 Expectancy awareness ratings

32 (58%) participants were classified as aware (15 males) and 19 as unaware (6 males). Aware and unaware groups did not differ significantly in terms of age or gender (see Table 1 for group characteristics).

**Fig. 2:**
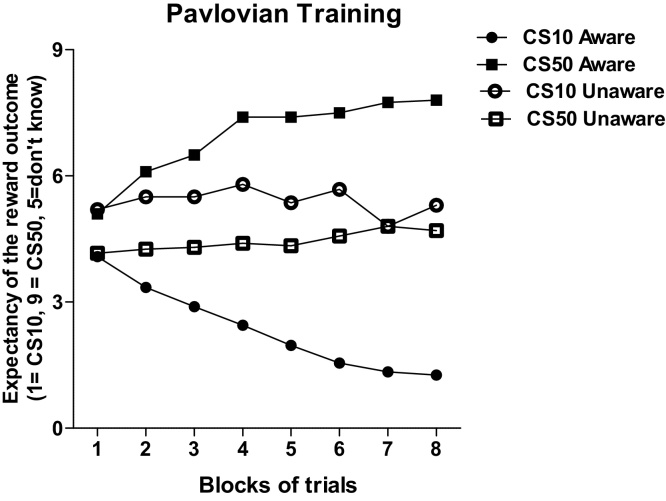
Mean expectancy of outcome during Pavlovian training for CS10 and CS50 trials across blocks of trials for aware and unaware participants.

Expectancy ratings showed a discrimination over time between the trials predicting a 50p and the trials predicting a 10p win, but only in the group of aware participants (a group x block x awareness interaction was significant [*F* (7, 343 = 11.5, *p *< 0.001, $\eta_{p}^{2}$ = 0.190], explained in a further ANOVA for aware and unaware groups separately by a CS by block interaction [*F*(7,217) = 34.45, *p* < 0.001, $\eta_{p}^{2}$ = 0.526] in the aware group but not in the unaware group ([*F*(7,126) = 1.21, *p *= 0.303, $\eta_{p}^{2}$ = 0.063; see Fig. 2). The post-hoc awareness questionnaire when the stimuli in each CS were presented in isolation showed that from the classified as aware participants (n = 32) at the end of the Pavlovian training, 30 participants were able to respond correctly about the predicted outcome when CS10 and CS50 were presented; one participant was able to respond correctly to CS10 and one participant to CS50 only. From the classified as unaware (n = 19), three participants were able to respond correctly to CS10 and four participant to CS50 only; twelve participants responded “I don’t know” to both stimuli. Thus post-hoc awareness identified 30 participants as fully aware, 12 participants as fully unaware and 9 participants as semi-aware.

#### 3.1.1 Emotional ratings (emotional conditioned response; ECR)

**Fig. 3:**
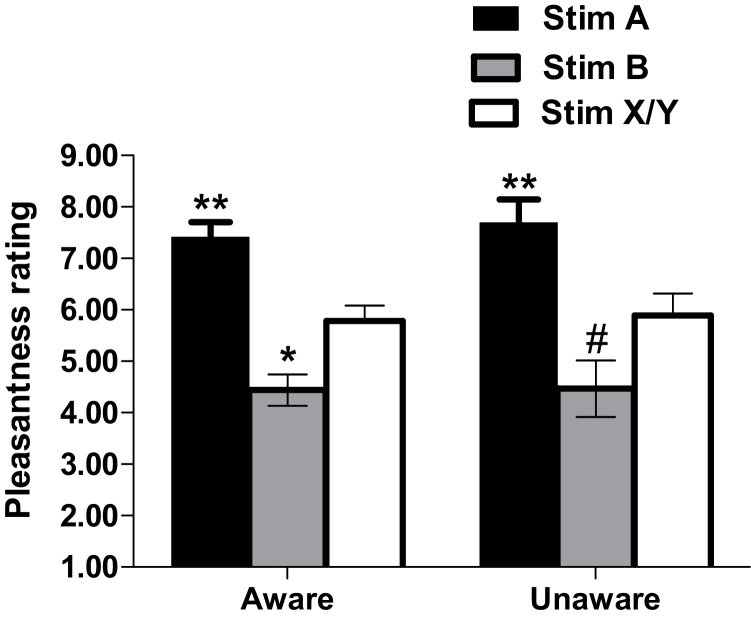
Mean pleasantness rating for stimulus A, B and X and Y combined (X/Y) following Pavlovian training for aware and unaware participants. ** P < 0.01 compared to ratings for stimulus B and X/Y. * p < 0.05 compared to X/Y in aware only participants; # p = 0.073 compared to X/Y in unaware participants.

In contrast to expectancy ratings there was no dissociation between the aware versus unaware group’s emotional ratings of each stimulus. For pleasantness ratings a mixed ANOVA was performed using stimulus A, stimulus B and stimulus X, Y combined as a within factor and awareness as a between factor. Awareness as factors confirmed a main effect of Stimulus [*F*(2,49) = 27.40, *p* < 0.001, $\eta_{p}^{2}$ = 0.359], with stimulus predicting 50p having higher pleasantness ratings than stimulus predicting 10p. Pleasantness ratings for stimuli X and Y combined were lower than the ratings of the stimulus predicting 50p and higher for the stimulus predicting 10p; see Fig. 3).

### 3.2 Instrumental training

**Fig. 4:**
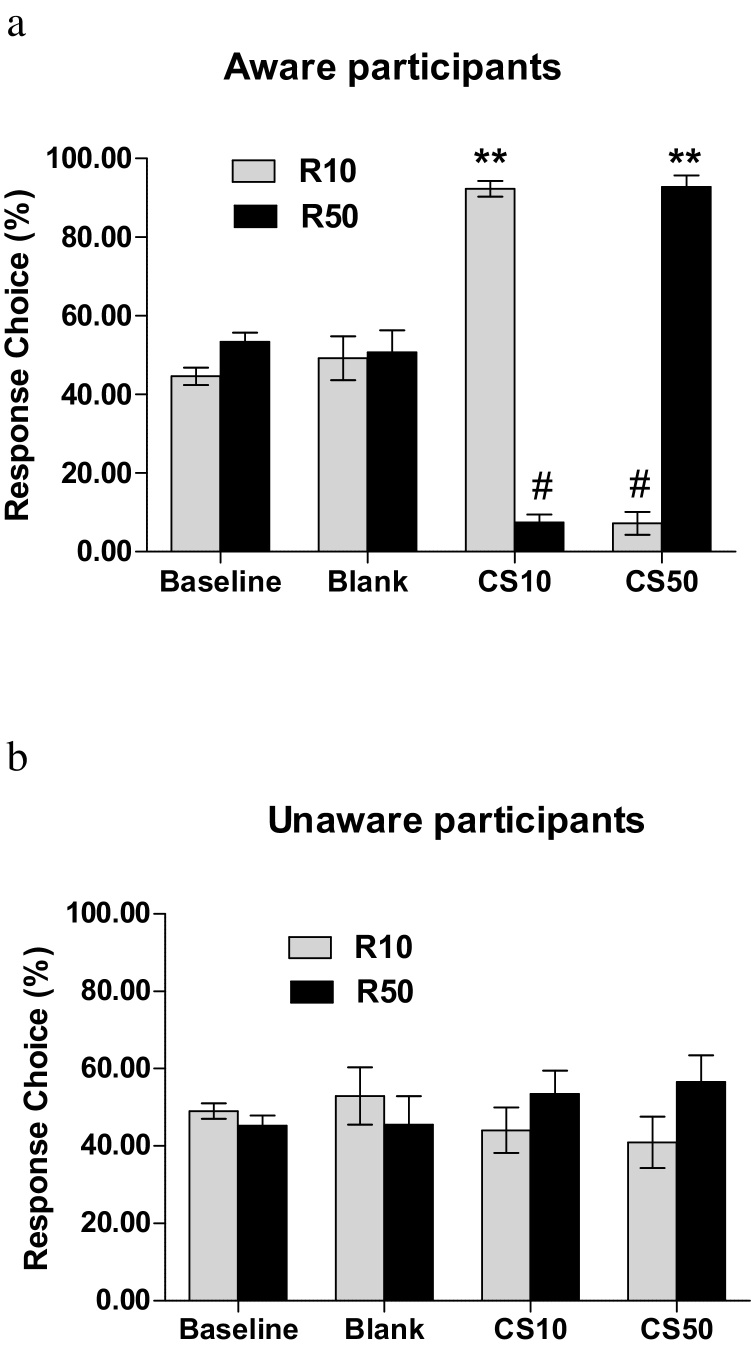
Mean Response Choice (R10 or R50) for aware [a] and unaware [b] groups during instrumental training (baseline) and during transfer when the conditioned stimuli were introduced for the blank, the CS10 and the CS50 trials. Trials of R10 response choice in the presence of CS10 and of R50 response choice in the presence of CS50 represent congruent trials; trials of R10 response choice in the presence of CS50 and of R50 response choice in the presence of CS10 represent incongruent trials; **; p < 0.001 compared to incongruent trial and to baseline and blank for both R50 and R10 response choices. #: p < 0.001 compared to baseline and blank for both R50 and R10 response choices. No significant differences were found for the unaware group (ps > 0.178). Congruent responses during CS10 and CS50 presentation were significantly different between aware and unaware (p < 0.001).

**Fig. 5:**
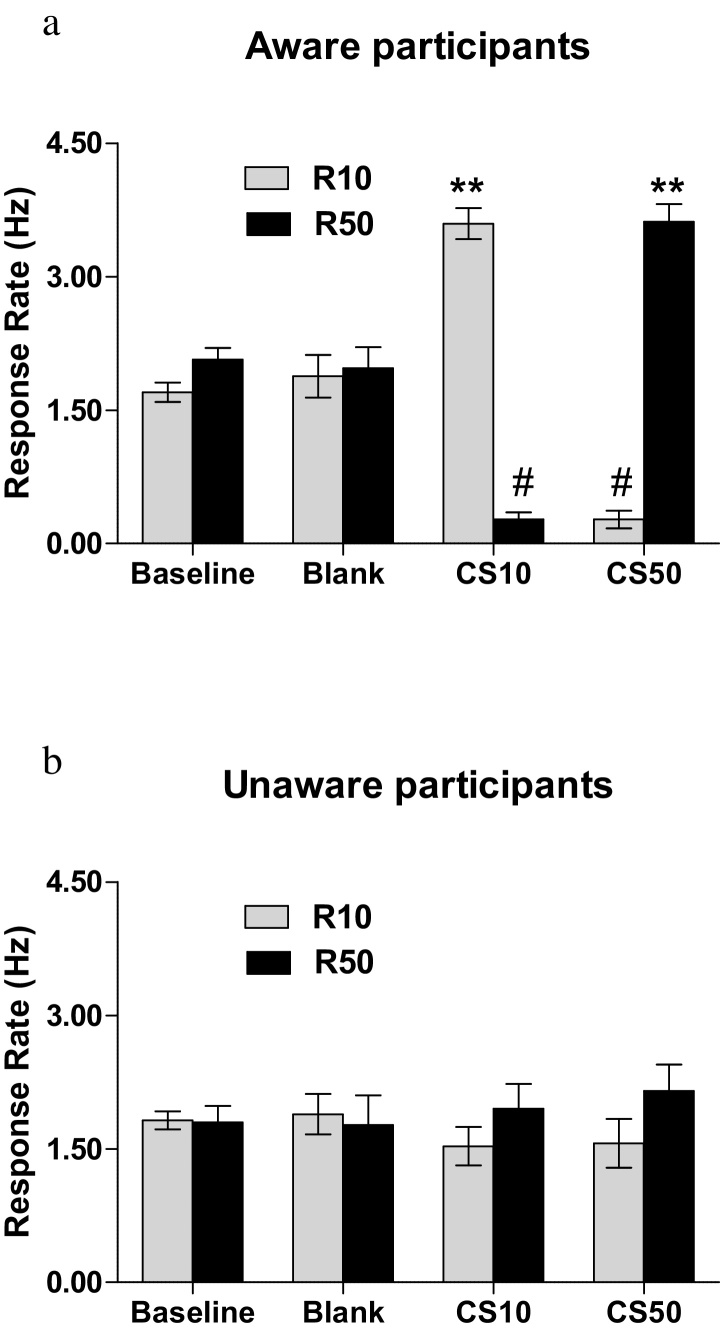
Mean Response Rate on Response keys (R10 or R50) for aware [a] and unaware [b] groups during instrumental training (baseline) and during transfer (when the conditioned stimuli were introduced) for the blank, the CS10 and the CS50 trials. Trials of R10 response choice in the presence of CS10 and of R50 response choice in the presence of CS50 represent congruent trials; trials of R10 response choice in the presence of CS50 and of R50 response choice in the presence of CS10 represent incongruent trials; ** p < 0.001 compared to incongruent trials and to baseline and blank trials for both on R50 and R10 number of responses (response rate). # p < 0.001 compared to baseline and blank for both on R50 and R10 number of responses (response rate). No significant differences were found for the unaware group (ps > 0.138). Congruent responses during CS10 and CS50 presentation were significantly different between aware and unaware (p < 0.001).

All participants, asked at the end of the experiment, correctly identified the causal relationship between each button and its monetary value. All participants’ response behaviour was comparable on each button by the end of training (i.e. there was no bias to either button). A mixed ANOVA on Response Choice (% of responses on R10 vs R50) and Response Rate (responses per second on R10 vs R50) for aware and unaware participants (entering awareness as a factor), found only that aware participants chose more often to respond overall than unaware participants [a main effect of Awareness was found for Response choice, [(*F*(1,49) = 9.98, p < 0.01 $\eta_{p}^{2}$ = 0.169]; no significant effects were found for response rates. Mean Response Choice and Response Rate (for R10 and R50 key) during instrumental training are given as baseline (before transfer phase started) for aware and unaware participants in Fig. 4, Fig. 5 respectively. Missing trials (trials during which participants decided not to press) varied between 1% and 16% with a mean of 4.5% (n = 39); all remaining participants chose to press in every trial.

### 3.3 Transfer

Nine participants did not chose to press at least in one of the trials; four participants did not press in trials when the blank was presented, seven when the CS10 was presented and three when the CS50 was presented. All remaining participants (n = 42) chose to press on every trial. Trials during which participants decided not to press varied between 3% and 34% with a mean of 9.5.

For response choice an Awareness x CS x Response key interaction [(F (2,98) = 37.67, p < 0.001, $\eta_{p}^{2}$ = 0.435],was explained in separate repeated measures ANOVAs for each awareness group, by a CS x response key interaction in aware [*F*(2,62) = 114.1, *p* < 0.001, $\eta_{p}^{2}$ = 0.786] but not in unaware participants [(*F*(2,36) = 1.11, p = 0.340 $\eta_{p}^{2}$ = 0.058; see Fig. 4a and b]. Post-hoc comparisons are given in the figures and figure legends 4a and 4b.

A similar pattern was observed for Response Rate. The mixed ANOVA found Awareness x CS x Response key interaction [*F* (2,98) = 33.53, *p* < 0.001, $\eta_{p}^{2}$ = 0.400]. The Awareness x CS x Response key interaction was investigated with separate repeated measures ANOVAs for each awareness group, with CS and Response key as factors. A significant CS x Response key interaction was found for the aware [*F*(2,62) = 95.0, *p* < 0.001, $\eta_{p}^{2}$ = 0.755] but not for the unaware group [*F*(2,62) = 0.654, *p* =0.526, $\eta_{p}^{2}$ = 0.035; see Fig. 5a and b]. Post-hoc comparisons are given in the figures and figure legends 5a and 5b

We also performed a mixed ANOVA for Response choice and Response rate, entering as an additional fixed factor Emotional Conditioned Response (ECR) group “high” versus “low” based on a median split of the differences in pleasantness ratings for CS50 over CS10. From the 32 aware participants 15 were in the group ECR “high” and 17 in “low”. From the 19 unaware participants, 10 were in the group ECR “high” and 9 in “low”.

The Awareness x CS x Response key interaction remained significant for both Response choice ([*F* (2.94) = 39.43, *p* < 0.001, $\eta_{p}^{2}$ = 0.456]) and Response rate ([*F* (2,94) = 35.42, *p* < 0.001, $\eta_{p}^{2}$ = 0.452]). No other relevant significant interactions involving ECR group were revealed. Table 2 presents Response choice and Response rates for the aware and unaware participants separately for participants with high and low ECR.

We repeated the ANOVA for Response choice and Response rate, entering as fixed factor ECR group “high” versus “low” but with post-hoc awareness as a fixed factor. For this analysis we divided our participants in aware (n = 30; the ones who responded correctly for CS10 and CS50) and unaware (n = 12; the ones who responded with “I don’t know”) based on post-hoc awareness ratings. The Awareness x CS x Response key interaction remained significant for both Response choice ([*F* (2,76) = 30.93, *p* < 0.001, $\eta_{p}^{2}$ *=* 0.449]) and Response rate ([*F* (2,76) = 25.54, *p* < 0.001, $\eta_{p}^{2}$ *=* 0.402]; data not shown). No other relevant significant interactions involving ECR group were revealed.

Correlations between pleasantness difference ratings for the stimulus CS50 over CS10 and Response rate differences for the CS50 over CS10 on their respective Response keys (congruent responses) were not significant either in aware (r=0.151, p=0.408) or in unaware (r=0.381; p=0.107) participants.

## 4 Discussion

The aims of the current experiment were twofold. One aim was to demonstrate that differential value of the outcome (50p versus 10p) would lead to an outcome specific transfer. Indeed participants increased their choices and rate of response for the respective response keys in the presence of CS50 and CS10. However this effect was seen only in participants aware of the CS-outcome contingencies based on outcome expectancy ratings. Another aim was to test whether a positive subjective emotional response elicited by a reward-paired cue could influence a separately trained reward-seeking response, independent of knowledge of the cue–reward association. The second hypothesis was not supported − a group of participants who displayed differential emotional responses to stimuli predictive of either 10p or 50p, despite an absence of explicit knowledge of these predictive relationships (the ‘unaware’ group), did not display differential behavioural responses when encountering the stimuli in an instrumental context. As mentioned above only participants who were aware of the CS–outcome associations displayed an influence of the CS on behaviour.

The present behavioural results are in accord with previous studies that have supported the role of expectancy awareness in mediating specific-PIT [8], [17], [26]. The current study strengthens this existing literature by finding that the emotional conditioned responses that accompany expectancy awareness do not by themselves elicit specific-transfer. Thus knowledge of reward availability is a necessary criterion in the control of reward-seeking choice by separately trained stimuli. Indeed, the magnitude of emotional conditioned response was indistinguishable in aware and unaware cohorts, thus any differences in behaviour cannot be attributed to differences in emotional reactivity. Moreover, the lack of specific-transfer in the unaware group was not due to ceiling effects constraining their behaviour − their overall Response Choice, i.e. response initiation, was subtly lower than the aware group, and Response Rate was no different. Instead, the distinction between awareness groups lay in their allocation of pressing to each button in the presence of each stimulus.

While the current data is supportive of the role of *expectancy* in *specific*-PIT, it is less able to explicate the role of *emotion* in *general*-PIT. The specific version is argued to rely on the stimulus activating the specific sensory identity of the outcome, which in turn *biases* choice of response towards that which procures the same outcome [5], [6], [27]. The general version is suggested to occur through the stimulus activating the general emotional features of the outcome, which in turn *augments* any concurrent response, regardless of whether stimulus and response share an outcome [12], [13]. The current experiment found no general augmentation of responding in the presence of the higher-reward CS50 compared to the lower-reward CS10, despite running additional analyses which included ‘high’ versus ‘low’ Emotional Conditioned Response as a factor.

However, this lack of general-PIT may be an artefact of the design employed. The present task used a specific-PIT paradigm which could not assess response augmentation (general-PIT) independently from response choice (specific-PIT), given that each CS served both as an emotional and a predictive stimulus. This may have biased participants towards using a more cognitive response selection strategy, which usurped any emotional response augmentation.

Nevertheless, the use of unaware participants in the current experiment may provide a valuable means to study general PIT if combined with an amended paradigm. For example, results from an alternative method provide a relatively consistent demonstration of general transfer in humans, and so may be more suited to the study of general-PIT [15], [16], [28], [29]; but see [30]]. The above authors used a modified PIT paradigm, whereby two stimuli (S1 & S2) predicted specific outcomes (O1 & O2) shared with two instrumental responses (R1 & R2), whereas a third stimulus (S3) was paired with an outcome (O3) that had no corresponding response, arguing that this precluded any specific-transfer effect in the presence of S3 [10]. Despite the apparent prevention of specific transfer, these studies still demonstrated an augmentation of general responding in the presence of the S3, and congruent biasing of response selection in the presence of S1 & S2. Moreover, rodent lesion studies have shown double dissociations of the brain regions necessary for general- and specific-PIT using this S3 method [10], [31], suggesting that the design may help delineate motivational structures in humans.

However, the S3 method has shown less consistent results in human brain imaging, with studies either not demonstrating general-PIT behaviourally [e.g. [30]], or not finding such clean dissociations neurally [32]. Similar to the present experiment, the lack of clear general-PIT may have been due to participants adopting a cognitive response strategy that usurped general response augmentation. Such cognitive strategies may be precluded by the selection of unaware participants, who have been shown by the present experiment to be unable to demonstrate specific-PIT, and so just as the S3 is argued to prevent specific-PIT, so too may the inclusion of unaware participants be used as an additional method to prevent specific-PIT.

Additionally, Nadler et al. [15] suggest that the heightened absolute value of ECR was key to their demonstration of S3 general-PIT. It is possible that the level of *appetitive* ECR in the present study was insufficient to arouse general-PIT, in comparison to the *aversive* ECR used by Nadler et al. However, Nadler et al. did not report measures of ECR. Thus, the present experiment provides a further procedure that can be integrated into future PIT studies with aware and unaware participants, in the form of simple evaluative ratings of Pavlovian stimuli, that may be able to further elucidate the role of ECR in general-PIT.

Whether behavioural sensitivity to ECR may be amplified by lacking conscious awareness of the conditioned stimulus is an important possibility that warrants exploration; if reward-seeking can be controlled by stimuli outside of conscious awareness, then behaviour may be less amenable to conscious control strategies. In a test of unconscious stimulus control, Pessiglione and colleagues used subliminal stimulus presentation to induce unawareness of the available outcome, and used discriminative instrumental paradigms where the stimulus signalled the utility of a response in gaining the outcome [33], [34]. Similar to the present results, Pessiglione and colleagues showed an emotional conditioned response to the reward-paired stimulus. But in contrast to the present data, they also showed greater instrumental responding to the reward-paired stimulus in their unaware participants. One explanation for the discrepancy between the behavioural results of Pessiglione et al. and those of the current study may be the use of concurrent versus separate S → O and R → O training. Whereas Pessiglione and colleagues’ design allowed a direct S → R association to form, the current experiment’s PIT design precluded such a direct association. Thus emotional conditioned responses in unaware individuals may influence behaviour if the stimulus has gained direct access to the response.

Although the development of emotional conditioned responses in the absence of expectancy awareness was not the focus of the present investigation, the data provide further support that emotional appreciation of a stimulus can occur in the absence of knowledge of its associated outcome [18]. While it is difficult to confirm the absence of awareness, Lovibond & Shanks [35] provide criteria for a robust study of awareness that the current procedure adhere to. Stimuli were abstract shapes that participants had not experienced before, therefore excluding external sources of emotion; a range of stimuli were used and their relationship to either outcome was counterbalanced, therefore precluding the confound that any one stimulus was intrinsically more emotional; expectancy awareness was tested during learning rather than during debriefing, thus reducing memory demands on the display of awareness.

Where the current design could be improved may be in the inclusion of an awareness measurement that more closely matches the evaluative conditioning measurement. Whereas expectancy was primarily assessed while associative learning was taking place and in the presence of the two stimuli (CSs, one predictive, the other non-predictive), emotion was assessed at the end of the conditioning and ratings were taken in the presence of a single stimulus (the predictive and non-predictive stimuli were presented separately). Difference in test conditions could be argued to reduce the sensitivity of the awareness measure, relative to the emotion measure [see 36], and so misclassify aware participants as unaware. However, given the post-transfer awareness data, which used individual stimulus presentation to classify participants, any misclassification was minimal. Only 2 participants originally classified as ‘aware’ failed to identify both CS → O relationships, and no originally ‘unaware’ participants were able to correctly classify both CS → O relationships post-transfer. Although 7 unaware participants were able to identify one of the CS → O relationships post-transfer, whether or not these 7 ‘unaware’, and 2 ‘aware’, participants were included in the analyses did not alter the pattern of results − only the aware group displayed specific-PIT. Thus, even if these 7 ‘unaware’ participants had gained some form of awareness prior to the transfer phase, their level of awareness was not sufficient to induce transfer.

Finally, although the current experiment finds that expectancy awareness is necessary for the control of behaviour by separately trained reward-paired cues, it does not attest to whether expectancy awareness is sufficient. It may be that knowledge of the outcome coupled with an emotional conditioned response is required, alternatively a cognitive representation alone may be all that is needed to influence reward-seeking.

The role of cognitive outcome representations [7], [11], [37] has been proposed, in that specific-PIT in both humans and rodents has been shown to be insensitive to outcome devaluation. Changes in outcome value have been shown to change the conditioned emotional response elicited by the outcome’s predictive stimulus [19], [20], [21]. But whether these changes in emotional conditioned response persist into specific-PIT contexts has yet to be confirmed. Future research should therefore measure emotional conditioned responses during transfer to assess whether specific-PIT is insensitive to both outcome value and conditioned emotional response.

To conclude, the present investigation tested the role of conditioned emotional response in PIT. Results showed that emotional response was not sufficient to elicit transfer, and instead that reward expectancy was necessary. While necessary in the current experiment, the sufficiency of reward expectancy in PIT has yet to be confirmed. Thus future research should manipulate conditioned emotional response during transfer, to test the behavioural effects of dissociated reward expectancy.

## Figures and Tables

**Table 1:** Age and Gender for the aware and unaware participants.

Measurements	Group Aware (N = 32)	Group Unaware (N = 19)	Statistics
Age	21.59 ± 3.87	21.42 ± 2.82	t(49) = 0.170; n.s.
Gender	M = 15; F = 17	M = 6; F = 13	χ^2^(1) = 1.15; n.s.

**Table 2:** Response choice and Response rate for each stimulus presented and each response key in transfer. Data (mean (SEM)) are presented separately for aware participants with low or high ECR and for unaware participants with low or high ECR.

	Aware (n = 32)		Unaware (n = 19)	
	Low ECR (n = 17)	High ECR (n = 15)	Low ECR (n = 9)	High ECR (n = 10)
Response Choice (%)				
Blank/R10	54.96 (8.2)	42. 71 (7.4)	76.74 (6.03)	31.56 (8.4)
Blank/R50	45.04 (8.2)	57.08 (7.5)	21.18 (5.7)	67.50 (8.1)
CS10/R10	92.36 (2.5)	93.33 (3.3)	50.69 (10.0)	38.13 (6.7)
CS10/R50	8.09 (2.6)	6.67 (3.3)	46.87 (10.7)	59.38 (6.0)
CS50/R10	9.37 (4.7)	4.7 (3.3)	47.57 (11.4)	35.00 (7.5)
CS50/R50	90.63 (4.6)	92.77 (2.9)	48.26 (12.0)	64.06 (7.2)

Response Rate (Hz)				
Blank/R10	1.94 (0.3)	1.82 (0.4)	2.42 (0.3)	1.42 (0.4)
Blank/R50	1.69 (0.3)	2.29 (0.3)	0.59 (0.2)	2.82 (0.4)
CS10/R10	3.30 (0.2)	3.93 (0.3)	1.53 (0.4)	1.53 (0.2)
CS10/R50	0.29 (0.1)	0.24 (0.1)	1.44 (0.4)	2.41 (0.3)
CS50/R10	0.35 (0.2)	0.17 (0.1)	1.55 (0.4)	1.57 (0.3)
CS50/R50	3.27 (0.2)	4.00 (0.3)	1.72 (0.6)	2.53 (0.2)
